# Low-grade inflammation in survivors of childhood cancer and testicular cancer and its association with hypogonadism and metabolic risk factors

**DOI:** 10.1186/s12885-022-09253-5

**Published:** 2022-02-09

**Authors:** Henrik Ekedahl, Sigrid Isaksson, Olof Ståhl, Karolina Bogefors, Patrik Romerius, Jakob Eberhard, Aleksander Giwercman

**Affiliations:** 1 Department of Oncology, Skåne University Hospital, Lund, Sweden; 2 Department of Clinical Sciences, Division of Oncology and Pathology, Lund University, Lund, Sweden; 3 Department of Clinical Sciences, Division of Pediatrics, Lund University, Lund, Sweden; 4 Department of Translational Medicine, Lund University, Malmö, Sweden; 5 Reproductive Medicine Center, Skåne University Hospital, Malmö, Sweden

**Keywords:** Cancer survivors, Inflammation, Hypogonadism, Metabolic syndrome, IL-6, IL-8, IL-10

## Abstract

**Background:**

In childhood (CCS) and testicular cancer (TCS) survivors, low-grade inflammation may represent a link between testosterone deficiency (hypogonadism) and risk of metabolic syndrome. We aimed to study levels of inflammatory markers in CCS and TCS and the association with hypogonadism and future cardio-metabolic risk factors.

**Methods:**

Serum levels of inflammatory markers and testosterone were analyzed in CCS (*n* = 90), and TCS (*n* = 64, median time from diagnosis: 20 and 2.0 years, respectively), and in controls (*n* = 44). Differences in levels between patients and controls were calculated using univariate analysis of variance. T-test and logistic regression were applied to compare levels of cardio-metabolic risk factors and odds ratio (OR) of hypogonadism and metabolic syndrome in low and high inflammatory marker groups after 4–12 years of follow up. Adjustment for age, smoking, and active cancer was made.

**Results:**

TCS and CCS, as compared to controls, had 1.44 (95%CI 1.06–1.96) and 1.25 (95 CI 1.02–1.53) times higher levels of IL-8, respectively. High IL-6 levels were associated with hypogonadism at baseline (OR 2.83, 95%CI 1.25–6.43) and the association was stronger for high IL-6 combined with low IL-10 levels (OR 3.10, 95%CI 1.37–7.01). High IL-6 levels were also associated with higher BMI, waist circumference, insulin, and HbA1c at follow up. High TNF-α was associated with higher diastolic blood pressure. No individual inflammatory marker was significantly associated with risk of metabolic syndrome at follow up. High IL-6 combined with low IL-10 levels were associated with risk of metabolic syndrome (OR 3.83, 95%CI 1.07–13.75), however not statistically significantly after adjustment.

**Conclusion:**

TCS and CCS present with low-grade inflammation. High IL-6 levels were associated with hypogonadism and cardio-metabolic risk factors. Low IL-10 levels might reinforce the IL-6 mediated risk of developing metabolic syndrome.

**Supplementary Information:**

The online version contains supplementary material available at 10.1186/s12885-022-09253-5.

## Background

The survival rates of childhood (CC) and testicular cancer (TC) have increased significantly during the last decennia, making long-term effects of these cancers and their treatment important issues to address. CC and TC survivors (CCS and TCS) have an increased risk of premature onset of chronic systemic disease, in particular metabolic syndrome and cardiovascular disease [1–3]. Also, hypogonadism is more frequently occurring in CCS and TCS than in controls [4]. Male hypogonadism is a syndrome defined as low testosterone combined with symptoms, such as fatigue, sarcopenia, weakness, depressed mood, and sexual dysfunction. Patients with hypogonadism are classified into primary hypogonadism (Leydig cell dysfunction, characterized by low testosterone and increased luteinizing hormone (LH)) and secondary hypogonadism (hypothalamic-pituitary failure, characterized by low testosterone and low or normal LH). A third category - compensated hypogonadism - has been implemented, encompassing patients with normal testosterone but elevated LH. It is considered as a possible sub-clinical state, that gradually might develop into a primary hypogonadism [5]. Hypogonadal TCS have an increased risk of metabolic syndrome [6]. In healthy men, secondary hypogonadism is associated with increased body mass index (BMI) [5, 7] and compensated hypogonadism is associated with increasing fasting glucose and diabetes [8] and has been identified as marker of increased risk of premature mortality [9]. The underlying mechanisms are however still uncertain.

Obesity and metabolic syndrome are associated with a chronic state of low-grade inflammation [10]. Furthermore, chronic inflammation, obesity, and insulin resistance are associated with low testosterone levels in men [11]. The associations between inflammation, metabolic risk factors, and hypogonadism are complex, and the causality is uncertain. There is evidence supporting bi-directional associations why impaired metabolic function and inflammation might both be the cause of and a result of hypogonadism [12–16].

Cancer and inflammation are tightly associated, and tumor-promoting inflammation has been recognized as an “enabling characteristic” of cancer development [17]. Increased levels of inflammatory markers have also been found in survivors of malignant diseases. Survivors of childhood acute leukemia had increased levels of leptin and interleukin-6 (IL-6) and had higher percent fat mass despite similar BMI as controls [18]. Sulicka et al reported increased levels of pentraxin-3, soluble vascular cell adhesion molecule-1, osteoprotegerin, and tumor necrosis factor (TNF)-related apoptosis-inducing ligand, whereas CRP, IL-6, IL-18, TNF-α, monocyte chemotactic protein-1, and soluble intercellular adhesion molecule-1 were unchanged in acute lymphocytic leukemia survivors [19]. Bandak et al showed that TCS with uncompensated Leydig cell dysfunction had higher CRP than eugonadal TCS [20].

To better understand the biological mechanism linking low-grade inflammation to testosterone deficiency as well as risk of cardio-metabolic disease, we studied the association between serum levels of inflammatory markers, hypogonadism, and cardio-metabolic risk factors. Our aims were to investigate if young male cancer survivors and controls differed in levels of low-grade inflammation, whether the level of inflammation was related to hypogonadism and if it was associated with cardio-metabolic risk factors.

## Methods

### Patients and controls

Patients from two patient cohorts, initially participating in studies on reproductive function and later re-invited for studies on testosterone levels in relation to cardio-metabolic risk factors and bone health, were retrospectively included.

**Fig. 1:**
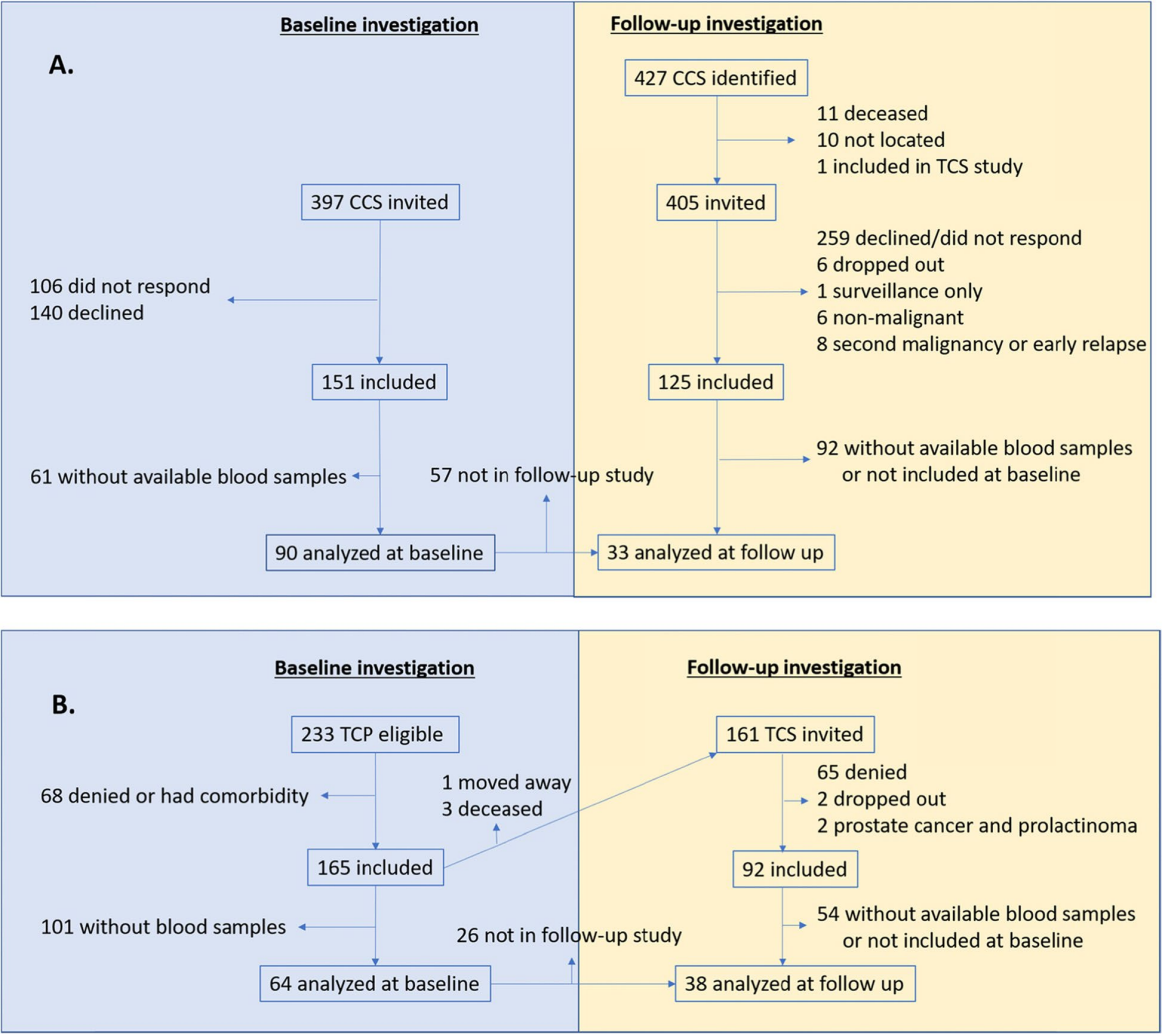
Flow charts of the inclusion of patients from the childhood cancer survivor cohort (**A**) and the testicular cancer patient cohort (**B**) in the present study

The CCS cohort was established in 2004 by inviting all men from the Region of Southern Sweden who were diagnosed with malignant disease or brain tumor before the age of 18 and reported to the Swedish Tumor Registry during 1970–2002. Further inclusion criteria were being 18–45 years at invitation and not receiving any oncological treatment for the last 4 years. Out of 397 eligible men, 151 were included in the study of reproductive function (Fig. 1A) [21]. In 2010 all CCS from the Region of Southern Sweden 1970–2002 were re-invited and 125 participants were included, with a partial overlap with the first study cohort (Fig. 1A) [4].

In the second cohort, 165 of 233 eligible TC patients (TCP) who were referred to the Department of Oncology, Lund University Hospital, Lund and accepted to participate in a study of reproductive function in TCP were included (Fig. 1B). Patients were below 50 years and diagnosed with TC < 5 years prior to inclusion and the study was open from March 2001 through October 2006 [22, 23]. At re-invitation in 2010 these TCS were aged 26–58 and 92 of 161 eligible patients were included (Fig. 1B) [4]. All patients, but one, were treated with unilateral orchidectomy. For testicular cancer patients, to different designations, TCP and TCS were applied since at baseline some of the patients had not yet completed cancer treatment and could, therefore, not be considered as TCS.

Blood samples from the baseline investigations (i.e. from the studies on reproductive function) were available from 90 CCS (median time from diagnosis to baseline: 20.4 years; range 6.7–36 years) and 64 TCP (median time from diagnosis to baseline: 2.0 years; range 0.0–5.0 years) and these men were included in the present study (Fig. 1A and B). Age and hormonal status at baseline are shown in Table 1. In the follow up study, 33 of 90 CCS (37%) and 38 of 64 TCP (59%, consequently called TCS onwards) were included, with a median follow up of 4.3 (range 3.7–5.5) and 8.9 (range 5.4–12.2) years, respectively. Comparing patients analyzed only at baseline and not at follow up with patients included in the follow up showed that patients not included at follow up were more often CCS (69% vs. 47%), had higher prevalence of hypogonadism (30% vs. 14%) and had higher levels of IL-6 (median 0.47 vs. 0.39 pg/mL, Table S1).

**Table 1:** Demographics of patients and controls at baseline

Characteristics	Testicular Cancer Patients *n = 64*	Childhood Cancer Survivors *n = 90*	Patients Total *n = 154*	Controls *n = 44*
**Age,** *mean (SD)*	34.6 (7.1)	30.5 (5.6)	32.2 (6.5)	36.0 (6.7)
**LH**^a,b^**,** *median (range)*	5.6 (2.0–23.0)	4.2 (1.1–15.0)	4.5 (1.1–23.0)	3.0 (1.0–8.2)
**TT**^a^**,** *mean (SD)*	13.1 (2.9)	15.8 (5.5)	14.7 (4.8)	15.6 (4.1)
**Gonadal status**^c^**,** *n (%)*				
Eugonadal wo TRT	46 (72)	67 (74)	113 (73)	43 (98)
Secondary HG wo TRT	4 (6)	9 (10)	13 (8)	1 (2)
Primary wo TRT	0 (0)	0 (0)	0 (0)	0 (0)
Compensated wo TRT	3 (5)	4 (4)	7 (5)	0 (0)
Eugonadal w TRT	1 (2)	8 (9)	9 (6)	0 (0)
Secondary HG w TRT	1 (2)	1 (1)	2 (1)	0 (0)
Primary w TRT	1 (2)	1 (1)	2 (1)	0 (0)
Compensated w TRT	1 (2)	0 (0)	1 (1)	0 (0)
Unknown w TRT	1 (2)	0 (0)	1 (1)	0 (0)
**Hypogonadal**^c^**,** *n (%)*				
No	46 (72)	67 (74)	113 (73)	43 (98)
Yes	12 (19)	23 (26)	35 (23)	1 (2)

^a^Data missing on 7 TCP. ^b^Data missing on 7 CCS. ^c^Data missing on 6 TCP. *LH* Luteinizing hormone, *TT* Total testosterone, *TRT* Testosterone replacement therapy, *wo* Without, *w* With

Controls in the same age range were previously identified through the Swedish Population Registry, to serve as healthy controls in a study of hypogonadism in subfertile men [24]. Participants with a history of malignant neoplasm or Klinefelter syndrome were excluded.

### Investigations at baseline

#### Hormone analysis

The procedure of hormone analysis is previously described [21, 23]. In summary, venous samples were drawn from patients between 8.00 a.m. and 15.00/16.00 p.m. for TCP and CCS at baseline. Serum testosterone levels were measured by using a luminometric technique (Access; Beckman Coulter, Chaska, MN, USA) since May 2001 and for some few patients included previously by using a RIA, an ‘in-house’ method developed at the department of clinical chemistry in Malmö. Conversion from RIA values to Access values was performed. Reference range for serum testosterone was 10–35 nmol/L.

For TCP, serum LH was measured by using an immunofluorometric assay (DELFIA LH, Wallac Oy, Turku, Finland) and after April 2000 by using the Elecsys LH immunoassay (Roche Diagnostics). A conversion between the methods was carried out. For CCS, LH was analyzed by using a two-step immunometric assay with a luminometric technique (Access; Beckman-Coulter), reference range 1.0–10 IU/L. Thus, reference ranges for TT and LH were the same for all methods enabling a uniform definition of hypogonadism.

Primary hypogonadism was defined as total testosterone (TT) < 10.0 nmol/L and LH > 10.0 IU/L. Secondary hypogonadism was defined as TT < 10.0 nmol/L and LH < =10.0 IU/L, and compensated hypogonadism was defined as TT > =10.0 nmol/L and LH > 10.0 IU/L. The definition of hypogonadism included patients with primary, secondary, and compensated hypogonadism as well as patients on testosterone replacement therapy (TRT).

#### Inflammatory assay

Inflammatory markers (IFN-γ, IL-1β, IL-2, IL-4, IL-6, IL-8, IL-10, IL-12p70, IL-13, and TNF-α) were analyzed in blood samples, stored in − 80 °C until analysis, from both patient cohorts and controls by using a V-PLEX Proinflammatory Panel 1 (human) Kit (MesoScaleDiscovery, Rockville, MD, USA), following the manufacturer’s protocol. Samples were run in duplicates and mean values were used. Samples from both patient cohorts and controls were run proportionally in each batch. For samples with a mean value below the lower detection limit, the value was set to 50% of the lower detection limit. For 4 of 10 inflammatory markers (IL-6, IL-8, IL-10, and TNF-α), <=5% of the samples were below the lower detection limit, whereas for the rest of the markers, > = 25% of samples were below lower detection limit (Fig. S1). Thus, we focused further analysis on IL-6, IL-8, IL-10, and TNF-α. Inflammatory markers were divided into two groups; ‘High’ – above the median of the controls, and ‘Low’ – below the median of the controls.

### Investigations at follow up

#### Metabolic parameters

The procedure of analyzing metabolic parameters is previously described [6]. At follow up, total cholesterol, high-density lipoprotein (HDL), low-density lipoprotein (LDL), and triglycerides were measured by using standard enzymatic methods. Plasma glucose was determined with an automated hexokinase method and serum insulin levels were measured by using an immunometric sandwich assay (Access Ultrasensitive Insulin, Beckman-Coulter, Brea, CA, USA). Blood levels of HbA1c (IFCC) were determined with the VARIANT TURBO Haemoglobin A1c Kit- 2.0 program (VARIANT TURBO, Hercules, CA, USA) using cat ion exchange and gradient elution. Insulin resistance was estimated using the Homeostatic Model Assessment for insulin resistance (HOMA-ir), calculated as (fasting insulin x fasting glucose)/22.5.

Measurements of weight, height and waist circumference were performed. In a supine position, brachial blood pressure in both arms and ankle blood-pressure were measured. The ankle-brachial pressure index (ABI) was defined as the ratio of the systolic blood pressure in the arms divided by the systolic blood pressure in the ankle. When the median brachial systolic pressure differed between the arms by more than 10 mmHg, the highest value was selected for ABI calculation.

The metabolic syndrome was defined using the NCEP ATP III criteria, which require three or more of following criteria; blood pressure > 130 mmHg systolic and/or > 85 mmHg diastolic, waist circumference > 102 cm, HDL < 1.03 mmol/L, fasting glucose > = 5.6 mmol/L, and triglycerides > = 1.7 mmol/L [25].

### Statistical analysis

The distributions of the concentrations of inflammatory markers were all positively skewed. Thus, natural logarithmic (Ln) transformation was applied, enabling statistical analysis for normally distributed variables (Fig. S1).

Differences in inflammatory marker levels between patients and controls were calculated using univariate analysis of variance and geometric means were used to describe the difference after back transformation. In order to exclude that levels of inflammatory markers mirror an active cancer those patients included 0–5 months (*n* = 10) from surgery, patients with stage IV disease (*n* = 3) and patients with this data missing (*n* = 3) were not included in the analysis. Since the mean age differed between patients and controls analyses of variance were adjusted for age.

In the analyses of the associations between inflammation and hypogonadism, levels of inflammatory markers were considered as independent and +/− hypogonadism (including those on TRT) as dependent variable. Binominal logistic regression was applied to calculate the odds ratio (OR) of hypogonadism for patients with levels of inflammatory markers above the medians of the controls. Adjustment for age and active cancer (as defined above) was performed.

For the analyses of differences in cardio-metabolic risk factors (BMI, waist circumference, triglycerides, HDL, LDL, cholesterol, hemoglobin, systolic and diastolic blood pressure, ABI, glucose, HbA1c, insulin, and HOMA-ir) at follow up according to levels of inflammation at baseline, t-test was used to calculate the difference in levels of risk factors between patients with levels of inflammatory markers above or below the medians of the controls. For non-normally distributed variables (triglycerides, insulin, and HOMA-ir), Ln-transformed data was analyzed. ORs for metabolic syndrome according to levels of inflammatory markers were calculated by binominal logistic regression and adjusted for active cancer, and age and smoking status at follow up. The inflammatory markers were analyzed separately except for analyses of ORs for hypogonadism and metabolic syndrome which were also calculated for patients with IL-6 levels above median combined with IL-10 levels below median of the controls.

Normally distributed variables and non-normally distributed variables are described as mean (SD) and median (range), respectively. IBM SPSS Statistics v.25 was used for analysis. *P*-values < 0.05 were considered significant.

## Results

### Levels of inflammatory markers in cancer survivors as compared to controls

Both TCP and CCS had elevated levels of IL-8, with geometric means that were 1.44 times (95% confidence interval (95%CI) 1.06–1.96, *p* = 0.022) and 1.25 times (95%CI 1.02–1.53, *p* = 0.032) higher than for controls, respectively. The differences remained statistically significant after adjusting for age (*p* = 0.014 and *p* = 0.007, respectively, Table 2). There was no statistically significant difference in levels of IL-6, TNF-α, or IL-10 between patients and controls.

**Table 2:** Median concentration (range) of inflammatory cytokines in testicular cancer patients without active cancer and childhood cancer survivors as compared to controls

Inflammatory marker	Controls ***n*** = 44	TCP ***n*** = 48	p	CCS ***n*** = 90	p
**IL-6**	0.44 (0.21–1.94)	0.42 (0.15–2.73)	0.688	0.46 (0.09–11.3)	0.560
**IL-8**	7.81 (2.56–67.0)	10.37 (3.94–465)	**0.014**	9.69 (2.50–91.5)	**0.007**
**IL-10**	0.22 (0.03–1.48)	0.19 (0.04–1.30)	0.580	0.21 (0.04–1.12)	0.727
**TNF-α**	2.25 (0.90–5.36)	2.00 (0.95–3.99)	0.086	2.25 (0.61–5.49)	0.468

*TCS* Testicular cancer patients, *CCS* Childhood cancer survivors, *IL* Interleukin, *TNF* Tumor necrosis factor

### Levels of inflammatory markers in relation to hypogonadism

At baseline, 35 patients (23%) were hypogonadal. High levels of IL-6 were associated with hypogonadism (OR 2.83, 95%CI 1.25–6.43, *p* = 0.013, Table 3). This was also significant when adjusted for age and active cancer (*p* = 0.019). There was no association between hypogonadism and levels of IL-8, IL-10, or TNF-α. However, combining a high IL-6 and a low IL-10 yielded an unadjusted OR of 3.10 (95%CI 1.37–7.01, *p* = 0.007) for hypogonadism. The result was significant also after adjustment for age and active cancer (*p* = 0.013).

**Table 3:** Odds ratio of hypogonadism in childhood cancer survivors and testicular cancer patients with levels of inflammatory markers at baseline below or above the median of controls (*n* = 148)

Inflammatory marker	Eugonadal patients *n* = 113 (76%)		Hypogonadal patients *n* = 35 (24%)		OR (CI) Unadjusted	OR (CI) Adjusted^a^
**IL-6**						
Low	60	(86)	10	(14)	ref.	
High	53	(68)	25	(32)	**2.83 (1.25–6.43)**	**2.73 (1.18–6.31)**
**IL-8**						
Low	35	(78)	10	(22)	ref.	
High	78	(76)	25	(24)	1.12 (0.49–2.59)	1.23 (0.52–2.92)
**TNF-α**						
Low	65	(80)	16	(20)	ref.	
High	48	(72)	19	(28)	1.61 (0.75–3.45)	1.77 (0.81–3.86)
**IL-10**						
Low	63	(72)	24	(28)	ref.	
High	50	(82)	11	(18)	0.58 (0.26–1.29)	0.60 (0.26–1.34
**IL-6 and IL-10**						
IL-6 low and/or IL-10 high	91	(82)	20	(18)	ref.	
IL-6 High + IL-10 Low	22	(59)	15	(41)	**3.10 (1.37–7.01)**	**2.89 (1.25–6.68)**

^a^Adjusted for age and active cancer. *OR* Odds ratio, *CI* Confidence interval

### Inflammatory marker levels and future cardio-metabolic risk factors

High IL-6 levels at baseline, as compared to low, were associated with higher BMI (mean 28.0 vs. 25.4 kg/m^2^, *p* = 0.017), higher waist circumference (mean 98.9 vs. 90.5 cm, *p* = 0.006), higher serum insulin (median 7.0 vs 5.0 mIU/L, *p* = 0.049), and higher HbA1c (mean 36.7 vs. 34.3 mmol/mol, *p* = 0.028, Table 4) at follow up. High TNF-α levels were associated with higher diastolic blood pressure at follow up (mean 83.2 vs. 77.7 mmHg, *p* = 0.021, Table 4). There was no significant difference in individual metabolic parameters in relation to levels of IL-8 and IL-10. No individual inflammatory marker was associated with risk of metabolic syndrome (Table 5). High IL-6 combined with low IL-10 levels were associated with risk of metabolic syndrome (OR 3.83, 95%CI 1.07–13.75, *p* = 0.039). However, this association was not statistically significant after adjusting for age, smoking status, and active cancer (*p* = 0.197).

**Table 4:** Metabolic risk factors in relation to unadjusted levels of inflammatory cytokines in survivors of testicular cancer and childhood cancer at follow up (*n* = 71)

Metabolic parameters	IL-6					IL-8					TNF-α					IL-10				
	Low		High		p	Low		High		p	Low		High		p	Low		High		p
**BMI**	25.4	(2.6)	28.0	(5.3)	**0.017**	25.6	(3.0)	27.0	(4.5)	0.181	25.8	(3.3)	27.7	(5.1)	0.061	26.2	(4.3)	27.0	(3.8)	0.415
**Waist circumference**	90.5	(8.2)	98.9	(14.3)	**0.006**	91.7	(9.5)	95.2	(12.8)	0.245	92.1	(10.4)	97.1	(13.6)	0.084	93.0	(12.0)	95.7	(11.6)	0.362
**Cholesterole**	5.1	(0.9)	5.3	(0.9)	0.274	5.4	(1.2)	5.1	(0.8)	0.288	5.1	(0.9)	5.3	(1.0)	0.496	5.2	(1.0)	5.1	(0.9)	0.898
**HDL**	1.2	(0.2)	1.2	(0.4)	0.542	1.2	(0.4)	1.2	(0.3)	0.631	1.2	(0.3)	1.1	(0.3)	0.330	1.2	(0.3)	1.2	(0.3)	0.372
**LDL**	3.3	(0.8)	3.5	(0.9)	0.348	3.5	(1.1)	3.4	(0.7)	0.544	3.4	(0.8)	3.5	(0.9)	0.472	3.4	(0.8)	3.5	(0.8)	0.480
**Triglycerides**	0.8	(0.2–5.2)	0.9	(0.3–3.8)	0.266	1.0	(0.2–5.2)	0.8	(0.3–3.8)	0.522	0.8	(0.2–5.2)	1.1	(0.4–3.8)	0.132	1.0	(0.2–5.2)	0.8	(0.3–2.0)	0.070
**Hemoglobin**	149.5	(9.3)	148.7	(7.0)	0.706	147.7	(10.1)	149.9	(7.4)	0.313	147.9	(8.2)	151.1	(8.6)	0.131	148.4	(9.1)	150.5	(7.0)	0.347
**Insulin**	5.0	(2.0–22.0)	7.0	(1.0–25.0)	**0.049**	5.0	(2.1–22.0)	5.0	(1.0–25.0)	0.368	5.0	(2.0–22.0)	5.5	(1.0–25.0)	0.351	5.0	(1.0–25.0)	6.0	(2.0–22.5)	0.220
**Glucose**	5.2	(0.5)	5.3	(0.5)	0.441	5.3	(0.5)	5.2	(0.5)	0.298	5.3	(0.5)	5.2	(0.5)	0.614	5.3	(0.5)	5.2	(0.4)	0.639
**HbA1c**	34.3	(4.0)	36.7	(4.5)	**0.028**	35.3	(3.2)	35.2	(4.8)	0.945	35.1	(4.2)	35.5	(4.6)	0.677	35.6	(3.6)	34.7	(5.6)	0.429
**Systolic BP**	128.4	(13.3)	128.2	(14.2)	0.933	127.1	(12.9)	128.9	(14.0)	0.596	127.0	(12.0)	130.5	(15.8)	0.298	128.4	(13.9)	128.2	(13.2)	0.945
**Diastolic BP**	77.9	(9.3)	82.3	(10.2)	0.065	78.1	(10.6)	80.7	(9.5)	0.297	77.7	(8.7)	83.2	(10.8)	**0.021**	78.9	(10.1)	81.3	(9.5)	0.333
**ABI**	1.1	(0.1)	1.1	(0.2)	0.296	1.1	(0.1)	1.1	(0.1)	0.332	1.1	(0.1)	1.1	(0.1)	0.592	1.1	(0.1)	1.1	(0.1)	0.909
**HOMAir**	1.1	(0.4–6.5)	1.6	(0.2–6.6)	0.095	1.1	(0.4–6.5)	1.2	(0.2–6.6)	0.417	1.1	(0.4–6.5)	1.2	(0.2–6.6)	0.597	1.0	(0.2–6.6)	1.3	(0.4–5.8)	0.162

*BMI* Body mass index, *HDL* High-density lipoprotein, *LDL* Low-density lipoprotein, *BP* Blood pressure, *ABI* Ankle brachial index, *HOMA-ir* Homeostatic Model Assessment for insulin resistance. Normally distributed variables are described by mean (SD), and non-normally distributed variables are described by median (range)

**Table 5:** Odds ratio for metabolic syndrome at follow up, unadjusted and adjusted for age, smoking status and active cancer, in survivors of testicular cancer and childhood cancer (*n* = 69)

Inflammatory cytokine	Metabolic syndrome				OR (CI) Unadjusted	OR (CI) Adjusted^a^
	Absent *n* = 54 (78%)		Present *n* = 15 (22%)			
**IL-6**						
Low	35	(85)	6	(15)	ref.	ref.
High	19	(68)	9	(32)	2.76 (0.85–8.94)	1.76 (0.45–6.88)
**IL-8**						
Low	17	(74)	6	(26)	ref.	ref.
High	37	(80)	9	(20)	0.69 (0.21–2.25)	0.57 (0.14–2.29)
**TNF-α**						
Low	36	(84)	7	(16)	ref.	ref.
High	18	(69)	8	(31)	2.29 (0.72–7.30)	4.12 (0.94–18.12)
**IL-10**						
Low	32	(73)	12	(27)	ref.	ref.
High	22	(88)	3	(12)	0.36 (0.09–1.44)	0.23 (0.04–1.22)
**IL-6 and IL-10**						
Others	46	(84)	9	(16)	ref.	ref.
IL-6 High + IL-10 Low	8	(57)	6	(43)	**3.83 (1.07–13.75)**	2.71 (0.60–12.38)

^a^Adjusted for active cancer, smoking status and age at follow up

## Discussion

In this follow up study of CCS and TCP we found, as compared to controls, statistically significantly higher serum levels of IL-8, but not of the three other inflammatory markers investigated: IL-6, IL10 and TNF-α. Hypogonadism at baseline, found in approximately one of four cancer survivors, was associated with higher IL-6 levels. IL-6 levels at baseline were also positively associated with waist circumference, BMI, HbA1c, and insulin levels at follow up. A high IL-6 combined with a low IL-10 was associated with an increased risk of metabolic syndrome. Together, the findings of this study support the role of inflammation in the increased morbidity risk in young male cancer survivors.

It is well documented that CCS and TCS have higher risk of metabolic and cardiovascular disease [1, 3, 26–28]. A possible contributor might be the increased prevalence of hypogonadism in CCS and TCS [4]. Testosterone levels were found to be inversely correlated with inflammatory markers in healthy men and in men with metabolic syndrome [11, 29]. Although testosterone seems to have an anti-inflammatory effect, the effect of TRT on inflammatory markers remains unclear (reviewed in [29]). In the present study, patients on TRT were included in the definition of hypogonadism and primary hypogonadism was not found in any man not given TRT. Notably, only 2 patients (1%) had fulfilled the biochemical criteria of primary hypogonadism despite ongoing TRT. However, 10% of young cancer survivors had secondary hypogonadism, this hormone deficiency being often linked to high waist circumference [12]. Increased visceral adiposity has been reported to lead to decreased testosterone levels, by yet not completely understood mechanisms, involving hypothalamic inflammation and decreased GnRH release [12] and/or obesity-related metabolic endotoxemia-induced IL-6 release, inhibiting testicular function [30]. Testosterone deprivation, on the other hand, increases body fat mass, which has been explained by an increase in subcutaneous, rather than visceral fat [12]. Visceral adipocytes represent an important source of IL-6 [31]. Thus, the fact that waist circumference, in the present study, was the metabolic parameter most strongly associated with IL-6, which in turn was the inflammatory marker associated with hypogonadism, speaks in favor of increased IL-6 levels being a key player linking visceral adiposity to secondary hypogonadism [32].

Although IL-6 levels were associated with waist circumference, BMI, HbA1c, and insulin levels the association with metabolic syndrome was not statistically significant. Neither was the association with other inflammatory markers statistically significant, although there was a trend towards increased risk of metabolic syndrome for patients with low IL-10 levels. Interestingly, a combination of high IL-6 and low IL-10 levels was associated with an increased risk of metabolic syndrome, although not statistically significantly after adjusting for confounders.

IL-10 is known as an anti-inflammatory cytokine, which has been proven to improve insulin sensitivity in mice [31]. In an Italian study, IL-10 levels were higher in obese women than in non-obese women, but were in both groups negatively associated with metabolic syndrome [33]. In a Dutch study, IL-10 capacity, measured as IL-10 levels after stimulus with lipopolysaccharide (LPS), was negatively associated with levels of glucose, HbA1c and LDL, as well as risk of type 2 diabetes, but not with BMI [34]. The results in our study suggest that IL-10 might counteract the negative effects of adiposity-related cytokines, such as IL-6, on insulin sensitivity, which is corroborated by a study in mice where IL-10 co-treatment prevented IL-6 induced insulin resistance [35].

IL-8 were significantly higher in CCS and TCP than in healthy control. IL-8 is a chemokine, excreted by normal and neoplastic cells, and was first recognized as a chemotactic factor of neutrophils. It is now also recognized as an important mediator of angiogenesis, which is a crucial process in promoting cancer progression and metastasis formation [36, 37]. Expression of IL-8 is induced by inflammatory signals, by several environmental factors (in particular hypoxia) and as a response to chemotherapy [37]. Interestingly, evidence of a stable increase in IL-8 expression in chemotherapy-treated cells, after growth in vivo, suggests that a paracrine mechanism might induce genetic or epigenetic changes [38]. In line with this theory, total body irradiation and hematopoietic stem cell transplant was associated with a distinct epigenetic signature in T cells, at genes controlling inflammatory process and oxidative stress [39]. Epigenetic modeling, e.g. via expression of miRNAs, does in fact regulate the secretion of cytokines [40]. As far as we know, increased levels of IL-8 have not been described previously in cancer survivors. In contrary, levels of IL-8 were reported to be lower in acute lymphoblastic leukemia patients as compared to controls, up to 1 year after completed treatment [41]. Simultaneously, levels of TNF-α and IL-2 were increased, whereas levels of IL-6 and IL-10 were the same as for the controls. Other studies have also shown presence of low-grade inflammation markers in cancer survivors. CRP has frequently been reported to be elevated in CCS and TCS [3, 42–44], conflicting results have been reported regarding IL-6 [18, 19, 42], whereas levels of TNF-α were reported at same levels for cancer survivors as in controls [18, 19, 42].

It has been proposed that advanced glycation end products (AGE), which are produced in response to oxidative stress, might induce low-grade inflammation, vascular damages and decreased antioxidant activity leading to a chronic low-grade systemic inflammation [44, 45]. Radiotherapy and several chemotherapy agents are known to cause oxidative stress and thus might cause increased levels of AGE, thus functioning as a “metabolic memory”, generating a vicious circle of formation of reactive oxygen species, oxidative stress, and inflammation. This hypothesis is supported by a study of total body irradiation-exposed ALL survivors having a seven-fold increase of AGEs compared to healthy controls [44]. Interestingly, IL-8 has been shown to be an intermediate factor in AGE-mediated cell signaling [46], suggesting a role for IL-8 in the process linking radio- and chemotherapy treatment in young patients to low-grade inflammation and risk of metabolic syndrome. IL-8 has previously been associated with metabolic syndrome [47] and exercise decreases levels of IL-8 in people with metabolic syndrome [48]. However, the association with metabolic syndrome was not corroborated in the present study.

This study was limited by the low number of patients, which made analysis of associations with different types of hypogonadism impossible. The lack of access to blood samples at follow up held us from exploring the development of inflammation in relation to hormone levels over time. Availability of anthropometric and metabolic parameters at baseline would also have provided better possibilities to study the directions of associations shown in this study. The analysis of inflammatory markers was performed by using a proinflammatory multiplex panel limited to 10 cytokines, leaving out other possibly useful markers, especially high sensitive CRP. Blood samples were drawn at slightly different conditions regarding fasting and timepoint during the day, which might affect the testosterone levels. However, we do not believe that this noise in study set up creates any systematic bias. It should be noted that patients not included in the follow up analysis had a higher prevalence of hypogonadism and higher IL-6 levels, compared to patients who were analyzed at follow up, and thus the incidence of metabolic syndrome among young cancer survivors might be underestimated in this study.

## Conclusion

Levels of IL-8 were increased in CCS and TCP compared to controls, indicating presence of low-grade inflammation in young male cancer survivors. An inflammatory pattern, characterized by increased IL-6, was associated with hypogonadism and adiposity. In addition, we provide evidence supporting the role of IL-10 as a counteracting response to IL-6 mediated inflammation, thereby preventing development of metabolic syndrome. Prospective studies are needed to fully clarify the mechanism linking early cancer, inflammation, hypogonadism, and risk of cardio-metabolic diseases. There is also a need of establishing standardized methods of analysis as well as establishing reference values. Future prospective studies could establish the role of inflammatory markers in the follow up of cancer patients and aid the design of preventive measures aiming to improve life quality and expectancy in young cancer survivors.

## Supplementary Information

**Additional file 1.** Supplementary tables and figures. Comparison of patients in and not in the follow up analysis, and distributions of inflammatory cytokine levels in survivors of testicular and childhood cancer and controls.

## Data Availability

The datasets generated and analysed during the current study are not publicly available since proper ethical permission for open data access has not been obtained, but are available from the corresponding author on reasonable request.

## Abbreviations

ABI: Ankle-brachial pressure index
BMI: Body mass index
CC: Childhood cancer
CCS: Childhood cancer survivors
CI: Confidence interval
IL: Interleukin
LH: Luteinizing hormone
OR: Odds ratio
TC: Testicular cancer
TCP: Testicular cancer patients
TCS: Testicular cancer survivors
TNF: Tumor necrosis factor
TRT: Testosterone replacement treatment
TT: Total testosterone
