# Supplementary material for: Low-grade inflammation in survivors of childhood cancer and testicular cancer and its association with hypogonadism and metabolic risk factors
Source: BMC Cancer. 2022 Feb 9;22:157. doi: 10.1186/s12885-022-09253-5 (PMC8827204; doi:10.1186/s12885-022-09253-5)
Supplement: Supplementary file 1 — Additional file 1. Supplementary tables and figures. Comparison of patients in and not in the follow up analysis, and distributions of inflammatory cytokine levels in survivors of testicular and childhood cancer and controls. [file 12885_2022_9253_MOESM1_ESM.pdf]

Supplementary tables and figures

Table S1. Comparison of patients in and not in the follow up analysis.

**Characteristics**	**Included at both investigations** *n=71*	**No follow up** *n=83*
**Cohort,** *n (%)*		
**TCS**	38 (53.5)	26 (31)
**CCS**	33 (46.5)	57 (69)
**Age,** *mean (SD)*	33.1 (6.5)	31.4 (6.5)
**LH,** *median (range)*	4.8 (1.2-15.0)	4.5 (1.1-23.0)
**SHBG,** *mean (SD)*	28.2 (9.0)	26.0 (10.9)
**Testosterone,** *mean (SD)*	15.5 (4.56)	14.1 (4.97)
**f-T,** *mean (SD)*	0.35 (0.11)	0.34 (0.12)
**Hypogonadal**[a]**,** *n (%)*		
No	56 (79)	57 (69)
Yes	10 (14)	25 (30)
**IL-6**	0.39 (0.05-2.73)	0.47 (0.09-11.26)
**IL-8**	9.50 (3.08-465)	9.81 (2.50-91.5)
**IL-10**	0.19 (0.04-1.30)	0.20 (0.04-1.03)
**TNF-α**	2.11 (0.96-3.99)	2.24 (0.61-5.49)

[a]Data missing on 6 patients.

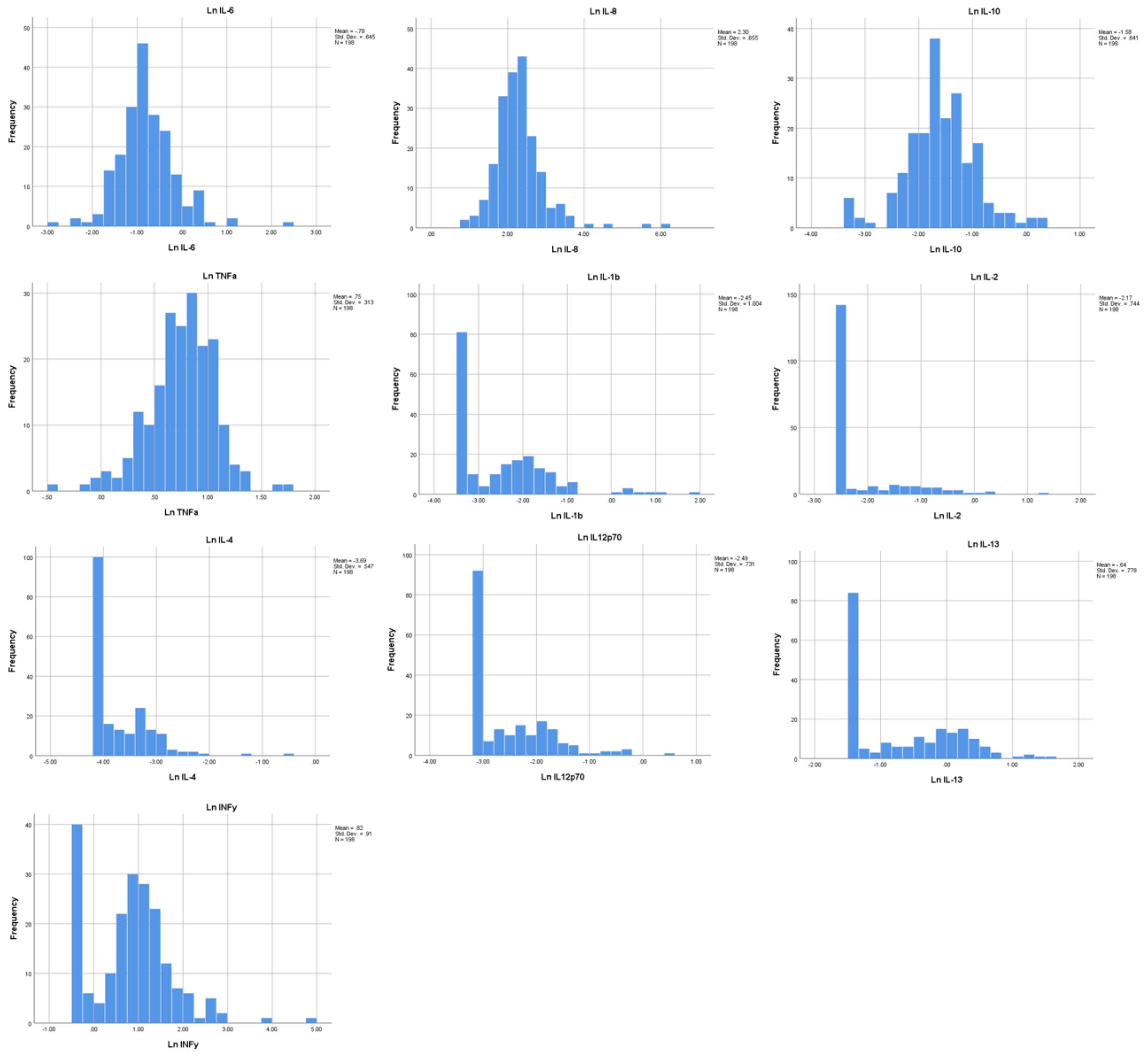

Figure S1. Distributions of inflammatory cytokine levels in childhood cancer survivors, testicular cancer patients and controls (n=198).
